# Amygdala lesions in rhesus macaques decrease attention to threat

**DOI:** 10.1038/ncomms10161

**Published:** 2015-12-14

**Authors:** Olga Dal Monte, Vincent D. Costa, Pamela L. Noble, Elisabeth A. Murray, Bruno B. Averbeck

**Affiliations:** 1 Laboratory of Neuropsychology, National Institute of Mental Health, National Institutes of Health, Bethesda, Maryland 20892-4415, USA

## Abstract

Evidence from animal and human studies has suggested that the amygdala plays a role in detecting threat and in directing attention to the eyes. Nevertheless, there has been no systematic investigation of whether the amygdala specifically facilitates attention to the eyes or whether other features can also drive attention via amygdala processing. The goal of the present study was to examine the effects of amygdala lesions in rhesus monkeys on attentional capture by specific facial features, as well as gaze patterns and changes in pupil dilation during free viewing. Here we show reduced attentional capture by threat stimuli, specifically the mouth, and reduced exploration of the eyes in free viewing in monkeys with amygdala lesions. Our findings support a role for the amygdala in detecting threat signals and in directing attention to the eye region of faces when freely viewing different expressions.

## Abstract (web summary)

The amygdala in the medial temporal lobe of the human and non-human primate brain is known to process salient social stimuli and to mediate threat discrimination. Here, Dal Monte *et al.* show that rhesus monkeys with amygdala lesions have deficits in detecting threat signals and directing attention to the eye region of a conspecific's face.

## Graphical abstract

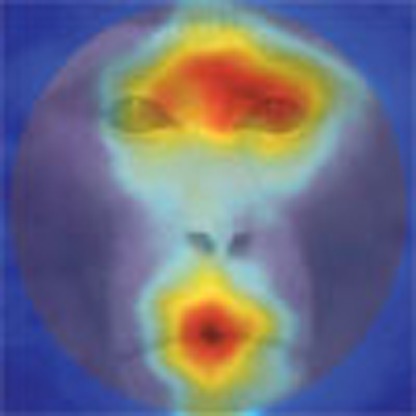

In both human and non-human primates, facial expressions are salient cues used as a means of non-vocal communication. The role of the amygdala in processing social stimuli, and specifically facial expressions, has been demonstrated by both imaging studies in human and non-human primates^1^,^2^ and studies in humans with amygdala damage^3^. Previous studies of human participants with amygdala lesions have contributed substantially to our understanding of the essential role of the amygdala in processing emotional facial expressions, particularly fear expressions^4^,^5^,^6^. More recently, several studies have suggested that the amygdala's role in evaluating emotional expressions extends to other threatening stimuli^7^,^8^,^9^. For example, studies in non-human primates have reported BOLD signal changes in the amygdala^10^ and increases in single-neuron activity^11^ for threatening faces relative to neutral and affiliative faces. This is consistent with a recent imaging study in human participants that showed significant signal changes in the amygdala for both fearful and angry faces^12^, suggesting a more general role for the amygdala in processing threatening and aversive stimuli.

Under free-viewing conditions, macaques, like humans, explore the eye region of the face more than any other facial feature^13^,^14^. In humans with amygdala damage, the deficits in perception and recognition of facial expressions can be attributed to decreased fixation on the eyes and increased fixation on the mouth while viewing faces^15^,^16^,^17^. Studies in monkeys have also shown that neurons in the amygdala respond preferentially to fixations on the eyes of conspecifics^18^, as well as during the production of expressions^19^. Despite strong evidence in humans and macaques that the eyes are a highly salient facial feature processed by the amygdala, it remains unclear if the amygdala specifically facilitates attention to the eyes or whether other features can also drive attention via amygdala processing.

To examine the contribution of the amygdala to processing facial expressions of emotion, we carried out two experiments. In each experiment, we compared the performance of four intact control macaques to four macaques that received selective, bilateral, excitotoxic amygdala lesions. The goal of the first experiment was to investigate attentional capture by images of facial features displaying different facial expressions. In the second experiment we examined gaze patterns of monkeys as they freely viewed images of whole conspecific faces displaying four different facial expressions. Together, the two experiments allowed us to characterize the effects of amygdala lesions on processing facial expressions of emotion. In addition, having within-subject data from the two experiments allowed for better comparison with previous literature in humans.

## Results

### Attention capture task

**Figure 1:**
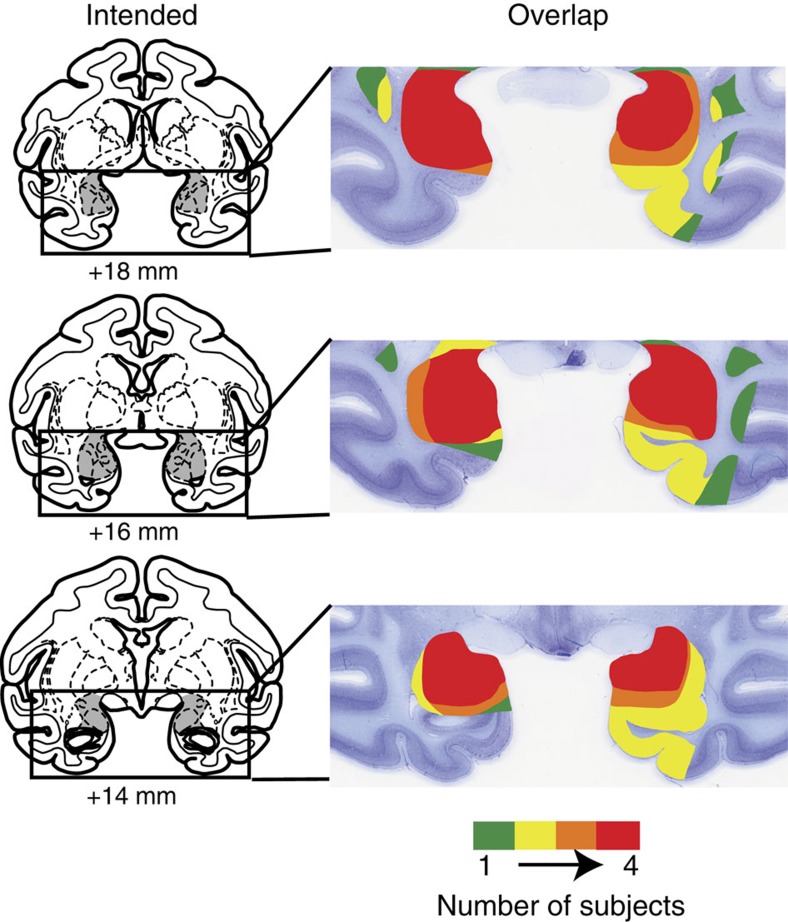
The left column shows MRI of coronal sections from a representative rhesus monkey brain depicting the location and extent of the intended bilateral amygdala lesion. The numerals indicate the distance (millimetres) of the sections from the interaural plane (0). The right column shows the location and extent of the bilateral amygdala lesions of four monkeys that received injections of excitotoxins in the amygdala at levels matching the sections in the left column.

**Figure 2:**
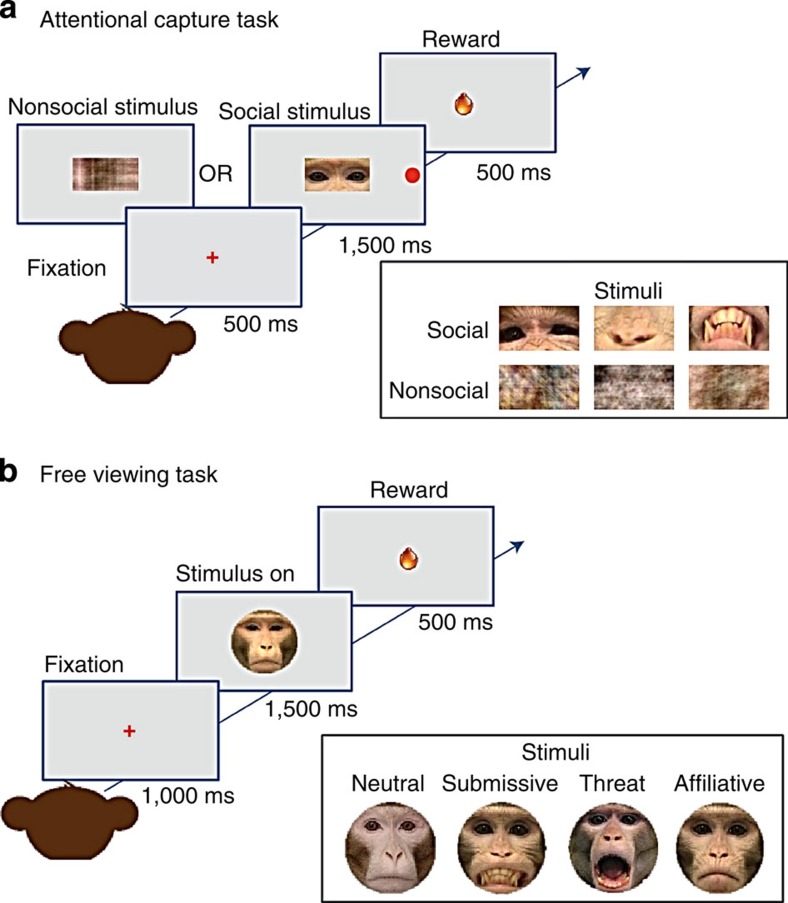
Behavioural tasks. (**a**) The attentional capture task. Animals faced a computer monitor on which fixation targets and social and nonsocial distractors were shown. After holding central fixation for 500 ms, a visual distractor image centred on the fixation point was simultaneously presented with a peripheral saccade target either to the left or right of center. When the animal correctly made a saccade to the peripheral target and held it for 500 ms, it received a juice reward. The visual stimuli presented at central fixation were either social (intact portion of a monkey face) or nonsocial (scrambled part of a face) images of a specific facial feature (eyes, nose or mouth) showing a particular facial expression (neutral, submissive, threat and affiliative). On the right are exemplars of the two different types of distractors shown; social and nonsocial. (**b**) The free-viewing task. Animals faced a computer monitor on which fixation targets was presented and monkeys initiated a trial by fixating a central fixation point for 1,000 ms. Following the initial fixation period a full picture of a conspecific face was shown for 1,500 ms. Faces appeared with either the mouth or a point mid-way between the eyes centred on the fixation point. This varied the initial position of the face relative to fixation. Monkeys were free to explore the face in front of them while an eye-tracking camera recorded their eye movements. At the end of the 1,500 ms presentation a juice reward was delivered, regardless of the gaze pattern of the subject. On the right are exemplars of the four different types of facial expressions shown (neutral, submissive, threat and affiliative).

Monkeys with amygdala lesions (*n*=4; Fig. 1) and unoperated controls (*n*=4) carried out an attentional capture task and a free-viewing task (Fig. 2). In the attentional capture task (Fig. 2a) monkeys were presented with a visual distractor image (social or nonsocial) replacing the central fixation point and a peripheral saccade target either to the left or right of center. The behavioural measure of interest was reaction time to initiate a saccade to the peripheral target. This allowed us to test the hypothesis that the social information displayed by the conspecific facial feature would capture attention within the context of ongoing goal-oriented behaviour. Because the images presented at fixation were irrelevant to the task, any effect of the centrally located social images on saccade latency, over and above that shown for the nonsocial images, can be considered attentional capture.

To assess overall attentional engagement, we investigated the number of errors (task performance, defined by failing to saccade when the central image and peripheral target were presented) made by both groups on this task. We found no effect of group (*F* (1, 6)=0.49, *P*=0.509), facial feature (*F* (2, 7.3)=2.13, *P*=0.188), stimulus type (*F* (1, 6)=4.66, *P*=0.074) or emotion (*F* (3, 15.2)=0.858, *P*=0.468) on the number of errors made. Also, there was no evidence of a higher-order interaction involving any of these factors.

**Figure 3:**
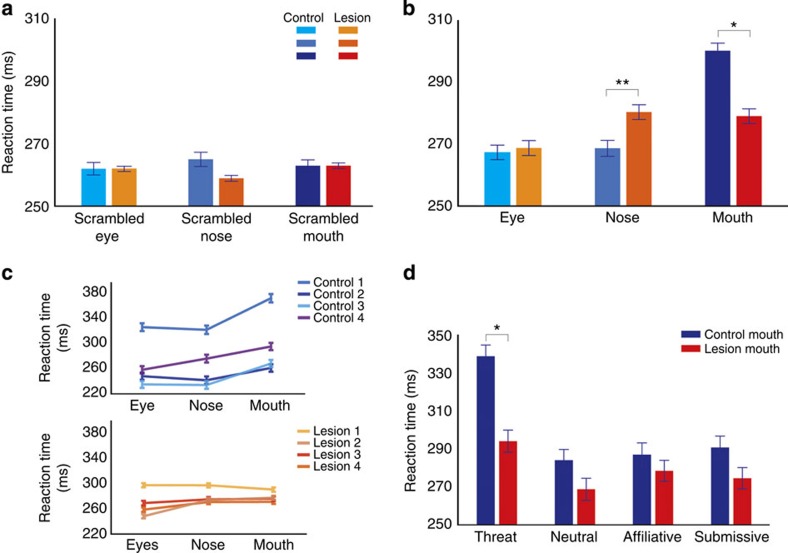
The effect of amygdala lesions on attentional capture by social stimuli. (**a**) Reaction time (ms) to saccade to the peripheral target for each group (four amygdala lesions and four controls) as a function of face feature (eyes, nose and mouth) for scrambled stimuli, collapsed across emotions. (**b**) Reaction time (ms) for each group (amygdala lesion and control) as a function of face feature (eyes, nose and mouth) for intact stimuli, collapsed across emotions. (**c**) Reaction time (ms) for each subject (top panel control and bottom amygdala lesion) as a function of face feature (eyes, nose and mouth) for intact stimuli. (**d**) Reaction time (ms) to saccade to the peripheral target for the intact mouth stimuli as a function of group (amygdala lesion and control) and emotions (threat, neutral, affiliative and submissive). Error bars in all plots represent ±1 s.e.m. *P<0.05, **P<0.01. These *P*-values are based on a mixed-effects ANOVA test.

We next investigated whether group (lesion and control), stimulus type (social and nonsocial), facial feature (eyes, nose and mouth) and emotion (neutral, submissive, threat or affiliative) influenced reaction times to saccade to the peripheral target during the task. Overall, we found that reaction times were longer when intact stimuli were presented at fixation relative to scrambled stimuli (Fig. 3a versus b, *F* (1, 33.7)=92.22, *P*<0.001). We then ran two separate analyses of variance (ANOVA), one for scrambled stimuli and one for intact stimuli. For scrambled stimuli there were no effects of group, facial feature or emotion on reaction time and there were no higher-order interactions (Fig. 3a; all *P*>0.05). In contrast, when social distractors were presented we found a significant interaction between group and facial feature (Fig. 3b, *F* (2, 68.6)=24.59, *P*<0.001) indicating that monkeys with amygdala lesions had faster reaction times than controls for some facial features. Thus, amygdala lesions attenuated the attentional capture effect produced by central presentation of social stimuli. However, one of the control animals had longer reaction times than the other animals (Fig. 3c). Therefore, we excluded that animal and ran the mixed-effects ANOVA on the intact facial features with three normal control and four amygdala lesion animals. Consistent with the effects when that animal was included, we found an interaction between group and face feature (*F* (2, 56)=22.4, *P*<0.001) and a significant face feature by emotion interaction (*F* (6, 168)=9.8, *P*<0.001). Thus, the effects were consistent with this animal removed from the analysis.

To further investigate differences between groups, we ran separate ANOVAs on the social stimuli for the eyes, nose and mouth. Trials in which the eyes were presented showed no evidence of significant effects of group or emotion (*P*>0.05). For trials in which the nose was presented we found a significant effect only of group, indicating that monkeys with amygdala lesions were slower than the controls (Fig. 3b, *F* (1, 32.8)=10.94, *P*=0.002). Finally, for trials in which the mouth was presented we found that controls had longer reaction times (Fig. 3b, *F* (1, 32.9)=5.03, *P*=0.032). The slower responses by controls also differed across the emotions (*F* (3, 104.9)=2.68, *P*=0.05). To isolate this interaction we examined the effect of group separately for each emotion for trials in which the intact mouth was presented (Fig. 3d). We found that controls were slower than the lesion group only for the threat emotion (*F* (1, 34)=6.3, *P*=0.017) but not for the other emotions (*P*>0.05). Thus, the threat expression on the mouth slowed controls more than animals with amygdala lesions.

We also examined whether monkeys with amygdala lesions were affected by the different emotions. We found that this group did show differential reaction times for different emotions (*F* (3, 51)=3.24, *P*=0.03), and different face features (*F* (2, 33)=10.48, *P*<0.001), as well as a significant interaction between emotion and face feature (*F* (6, 478)=2.53, *P*=0.019). We ran an additional ANOVA for each face feature. In trials in which the eyes or nose were presented we found no evidence of significant effects of emotion (all *P*>0.05). For trials in which the mouth was presented there was a main effect of emotion (*F* (3, 50.93)=4.41, *P*=0.008). *Post hoc* tests showed that monkeys with amygdala lesions were slower when presented with a threat mouth compared with neutral (*P*=0.001) or affiliative mouths (*P*=0.002). The other comparisons were not significant (*P*>0.05). Thus, the monkeys with amygdala lesions were affected by the emotions, although the effect was significantly attenuated compared with controls.

### Free-viewing task

The same subjects were evaluated in a free-viewing paradigm (Fig. 2b). The behavioural measure of interest was looking-time, expressed as the proportion of time that monkeys spent exploring each of the areas of interest (AOIs) compared with overall exploration. We included all successful trials, defined as maintaining fixation for the initial 1,000 ms fixation period before the faces were shown, even if the animals did not look at the face displayed after the initial fixation period.

**Figure 4:**
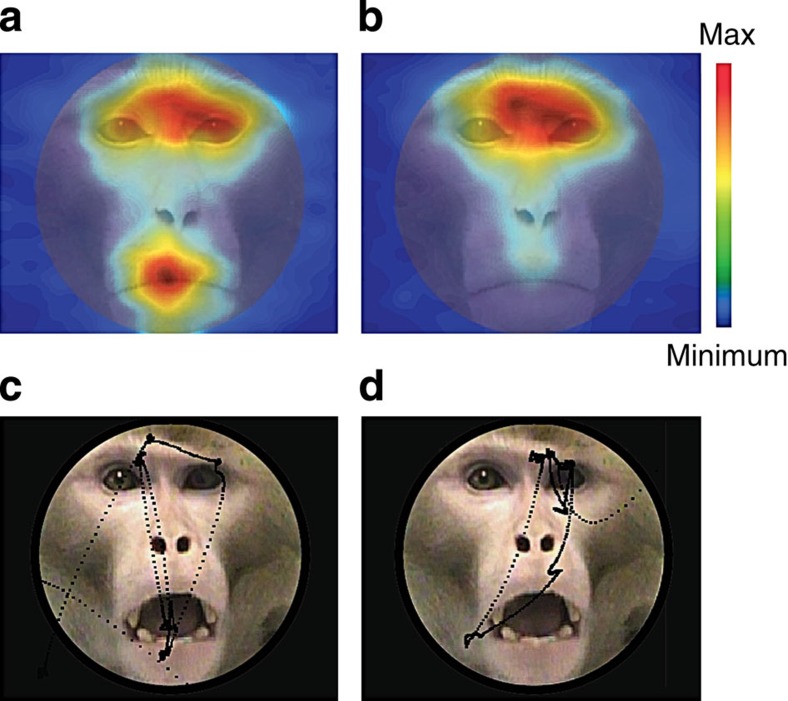
Fixation density plots. Colours indicate normalized looking-time. (**a**) The pattern of eye movements in animals with amygdala lesions. (**b**) The pattern of eye movements in unoperated contros. Panel illustrates pattern of eye movement where red indicates longer exploration time, blue indicates less; overlaid on example monkey face. All plots are averages per monkey (four amygdala lesions and four controls) across expressions. (**c**) An example of an amygdala lesioned monkey's eye movements. (**d**) An example of a control monkey's eye movements.

**Figure 5:**
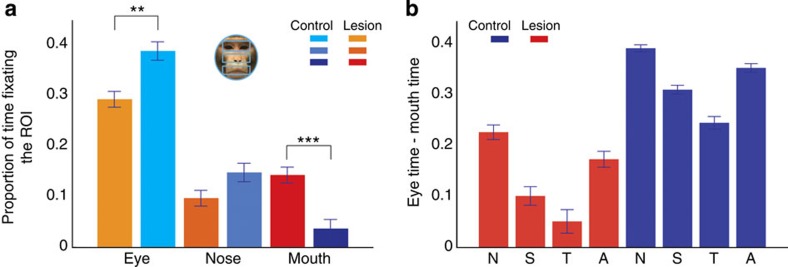
Decreased attention to the eyes in monkeys with amygdala lesions. (**a**) One example of the three AOIs superimposed on the neutral expression used for the analysis; eyes, nose and mouth. Proportion of looking-time to the eyes, nose and mouth AOI as a function of group (amygdala lesion and control). (**b**) Proportion of looking-time at the eyes minus looking-time at the mouth as a function of group (amygdala lesion and control) and emotion (neutral, submissive, threat and affiliative). Error bars in all plots represent ±1 s.e.m. **P<0.01, ***P<0.001. These *P*-values are based on a mixed-effects ANOVA test.

We began by comparing fixation density plots (Fig. 4) for the two groups. Visual inspection of the plots suggested that the control group spent more time fixating the eye region than any other part of the face (Fig. 4b), whereas the group with amygdala lesions split their time more evenly between the eyes and the mouth (Fig. 4a). Individual example trials also reflected these preferences (Fig. 4, bottom). To characterize this in more detail, we calculated total looking-time in three AOIs: the eyes, the nose and the mouth (Fig. 5a, inset). We then examined whether group (lesion and control), initial face position (eye centred and mouth centred) and emotion (neutral, submissive, threat or affiliative) influenced the proportion of time spent within each AOI. We found a main effect of AOI (Fig. 5a, *F* (2, 64)=137.5, *P*<0.001). *Post hoc* tests revealed that monkeys viewed the eyes more than the nose (*P*<0.001) or the mouth (*P*<0.001). We also found significant interactions between region and group (Fig. 5b, *F* (2, 64)=26.4, *P*<0.001). However we did not find a significant three-way interaction between region, group and emotion (*F* (6, 199)=0.85, *P*=0.535) nor a four-way interaction between region, group, emotion and initial face position (*F* (6, 203)=0.85, *P*=0.471). To further explore the region by group interaction we ran three separate ANOVAs, one for each AOI to investigate the effect of group (Fig. 5a). Consistent with the fixation density plots, we found that monkeys with amygdala lesions explored the eyes less than controls (*F* (1, 32)=9.24, *P*=0.005) and explored the mouth more than controls (*F* (1, 32)=61.77, *P*<0.001). There was no group difference for looking-time for the nose region (*P*>0.05).

Finally, we ran separate ANOVAs for each group, to investigate the effect of emotion and region (Fig. 5b, *F* (6, 199)=22.2, *P*<0.001). For the control group we found a main effect of region (*F* (2, 38)=58.44, *P*<0.001) and a significant interaction between region and emotion (*F* (6, 115)=9.47, *P*<0.001). Intact monkeys spent less time looking at the eyes when viewing threat faces compared with neutral (*P*<0.001) or affiliative (*P*<0.001) faces. Consistent with this finding, controls explored the mouth more for threat expressions than for any other emotion (all *P*<0.001).

For the amygdala lesion group we found an effect of region (*F* (2, 38)=10.63, *P*<0.001), and a significant interaction between emotion and region (*F* (6, 123)=13.6, *P*<0.001). *Post hoc* comparisons for each AOI showed that, similar to the controls, monkeys with amygdala lesions spent less time fixating in the eye region when viewing threat faces compared with neutral (*P*<0.001) or affiliative (*P*<0.001) faces, and more time exploring a threat mouth relative to a neutral (*P*<0.001) or affiliative (*P*<0.001; Fig. 5b) mouth. Thus, monkeys with and without amygdala lesions showed a similar exploration pattern with respect to expression; when presented with a threat image they looked longer at the mouth and less at the eyes compared with the other emotions. The lesion group still explored different face regions and expressions, but the effect of the expression on looking-time was diminished compared with controls (Fig. 5b).

### Pupil dilation during the free-viewing task

**Figure 6:**
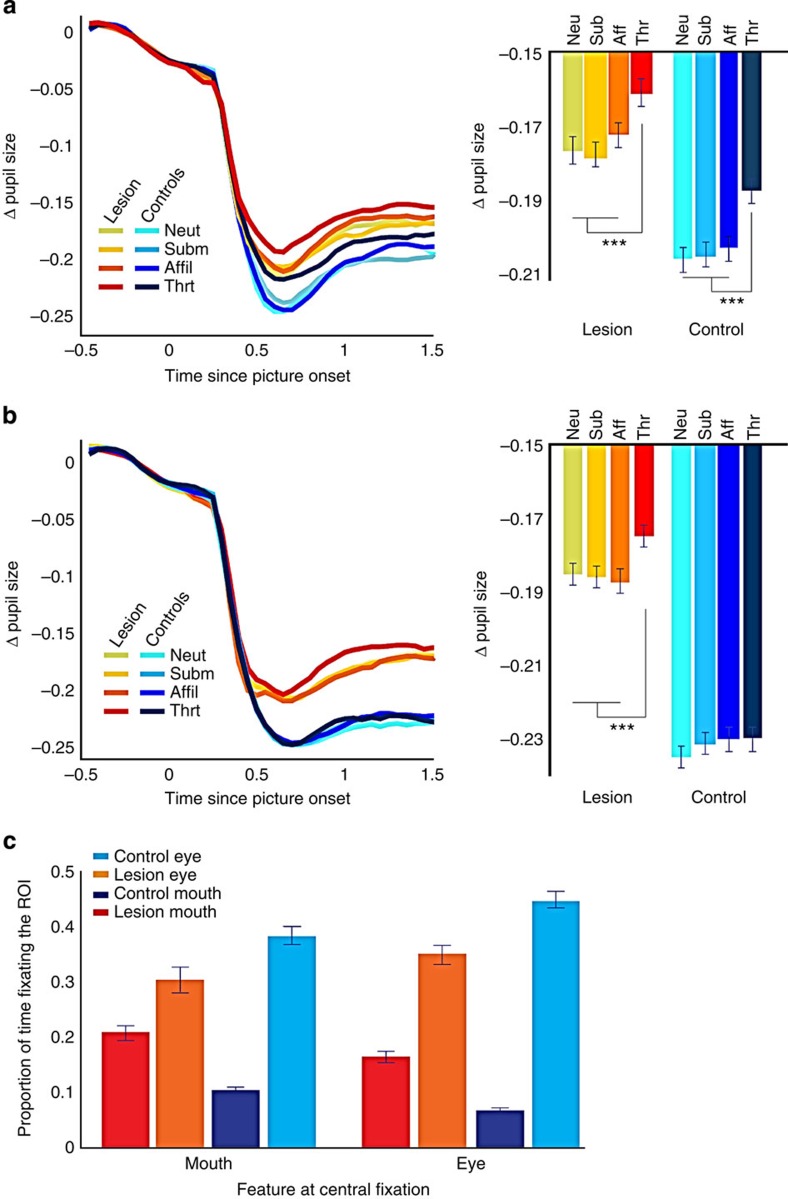
Emotion modulates changes in pupil dilation depending on the facial features that are fixated. (**a**) Pupil size, deviated from the baseline fixation period, for the lesion and control groups broken out in terms of the facial expression viewed by the monkeys, when vertical position of the stimulus was oriented so that the mouth appeared at central fixation. (**b**) The same as **a** but when the vertical position of the stimulus was oriented so that the eyes appeared at central fixation. (**c**) The proportion of time monkeys with or without amygdala lesions spent looking at the eye or mouth region as a function of which feature appeared at central fixation. Error bars in all plots represent ±1 s.e.m. ***P<0.001. These *P*-values are based on a mixed-effects ANOVA test.

We also examined changes in pupil size in the free-viewing task as a metric of arousal. The initial onset of the stimulus elicited a pupillary light reflex that spanned the first 500 ms of each trial (Fig. 6a,b). We found main effects of group (*F* (1, 32)=54.59, *P*<0.001) and emotion (*F* (3, 96)=22.1, *P*<0.001). On average, monkeys with amygdala lesions exhibited a smaller light reflex compared with controls. In addition, the mean change in pupil size during free viewing indicated greater dilation when viewing threat faces compared with neutral (*F* (1, 32)=87.2, *P*<0.001), submissive (*F* (1, 32)=46.6, *P*<0.001) or affiliative faces (*F* (1, 32)=27.9, *P*<0.001). However, it was evident that greater pupil dilation when monkeys viewed threat faces varied between groups as a function of the facial feature that appeared at central fixation (*F* (3, 32)=3.45, *P*=0.019). When the mouth appeared at central fixation (Fig. 6a), both controls (*F* (3, 48)=11.97, *P*<0.001) and monkeys with lesions (*F* (3, 48)=13.39, *P*<0.001) showed greater pupil dilation when they viewed threat faces compared with when they viewed other facial expressions. By contrast, when the eyes appeared at central fixation (Fig. 6b), group differences emerged (*F* (3, 96)=3.49, *P*=0.019). Specifically, greater pupil dilation to threat faces was observed in monkeys with amygdala lesions (Fig. 6b, *F* (3, 48)=9.38, *P*<0.001) but not in controls (*P*>0.05). This pattern is substantiated by the looking-behaviour of the two groups as a function of facial feature that appeared at central fixation. Both groups explored the mouth longer when it appeared at central fixation, compared with the eyes (Fig. 6c, *F* (2, 96)=21.9, *P*<0.001). Also, as already noted, the animals with amygdala lesions looked longer at the mouth and less at the eyes than control monkeys. Therefore, the difference in pupil dilation between groups when initial fixation was on the eyes may be due primarily to differences in the amount of time spent viewing the mouth.

## Discussion

We examined the effects of amygdala lesions on attentional capture by social and nonsocial stimuli, as well as gaze patterns and changes in pupil dilation when free viewing different facial expressions. Using the attentional capture task we found that monkeys with amygdala lesions and controls had similar reaction times when they were presented with nonsocial images (scrambled images of face features) at fixation. Notably, however, the groups differed when the monkeys were presented with social images (intact images of facial features). Specifically, relative to controls, monkeys with amygdala lesions showed faster reaction times to saccade to a peripheral target when the mouth portion of a threat face was presented. Using a free-viewing paradigm, we found that all monkeys, controls and operated alike, viewed the eye region more than any other region of the face. Relative to controls, however, monkeys with amygdala lesions spent less time exploring the eye region and more time exploring the mouth. In addition, both groups spent relatively more time exploring the mouth region and less exploring the eyes when viewing threat expressions compared with other emotions. When changes in pupil size were examined, we found greater pupil dilation for both groups when the animals viewed threat faces.

Across both groups the attentional capture effects on reaction times were driven by the mouth from threat faces. Other studies using related paradigms in intact monkeys have also found attentional capture effects specific to threat faces^20^. In a recent study, Bach *et al.*^21^ examined the effect of emotion on visual search efficiency using the face-in-the-crowd task in two human participants with amygdala lesions. Patients detected a happy face target faster than an angry face target, whereas the reverse was true for normal subjects. Together these results emphasize the important contribution of the amygdala in driving attention to threat stimuli.

Group differences in the attentional capture task were found with intact but not scrambled social stimuli. This is consistent with previous findings in human studies that have shown specific effects of processing emotional stimuli in participants with amygdala lesions. For example, subjects with amygdala damage were not impaired compared with controls when performing a spatial-cueing task when the orienting cue was a directional arrow, but were impaired when the cued orientation needed to be inferred by the eye gaze direction^22^. In addition, relative to controls, human subjects with bilateral amygdala damage show less attentional modulation for aversive compared with neutral words^23^.

The group differences in the attentional capture task were specific to the threat expression, and specifically the mouth. In these trials controls were slowed by about 60 ms relative to the other emotions, whereas animals with amygdala lesions were only slowed by about 15–20 ms. There were no group differences for the eyes. In rhesus monkeys, the expressive differences in the eye region are less dramatic than those in the mouth region. Macaque monkeys have large canine teeth, and display them prominently in some expressions, which may explain why the mouth captured attention more than the eyes in this paradigm. Furthermore, the stimuli used in our experiment depicted adult male monkeys with whom the test subjects were unfamiliar. Because of the unfamiliarity of the conspecific faces, the threat mouth images in the attentional capture task might be perceived as an important signal of potential danger. The attentional capture effects, therefore, may be due to the salience of the mouth as a sociobiological cue. In humans, the eyes and eyebrows contain cues that differentiate expressions^15^,^24^, whereas in monkeys the mouth appears to convey more information, particularly for threatening and submissive expressions^25^. It should be noted that angry and fearful faces represent qualitatively different forms of threat in the environment. While fearful facial expressions signal the presence of threat or danger, angry facial expressions signal a more direct and immediate threat towards the observer. Öhman^26^ suggests that aggressive facial expressions constitute the prototypical stimulus for eliciting social submission, such that vulnerable, low-ranking, socially submissive animals show increased anxiety when faced with aggressive displays. Therefore, the effect of the threat mouth found in our data may be due to the overt nature of aggression displays in this face region and elicit social submission.

Our findings from the attentional capture task suggest two conclusions. First, our results underscore a causal contribution of the amygdala to threat detection, even when that threat is irrelevant to ongoing goal-directed behaviour. Consistent with this, a functional magnetic resonance imaging (fMRI) study^27^ revealed amygdala activation to both attended and unattended images of fearful faces and the magnitude of the amygdala activation to unattended fearful faces was a function of an individual's level of anxiety. Second, our results indicate that even when damage to the amygdala is complete, or nearly so, there remains some capability for threat detection. Thus, it appears that neural circuits outside the amygdala contribute to marshalling the response to threat, at least in the social domain.

Research investigating the components of attention in normal individuals has established that threat can modulate both attentional engagement and disengagement (defined as the level of difficulty in shifting attention away from threat)^28^,^29^. Furthermore, high-trait anxious individuals show both impairment in disengaging from threat and no attentional capture by threat stimuli. Our paradigm was not designed to distinguish between these two mechanisms, and future studies are needed to tease apart how the amygdala contributes to either process.

We further investigated the role of the amygdala when animals freely viewed conspecific faces displaying different expressions. Consistent with previous studies in monkeys^13^,^14^ and human participants^17^, both groups spent more time exploring the eyes than the mouth. However, control animals spent more time exploring the eye region compared with monkeys with amygdala lesions, whereas the lesion group spent more time exploring the mouth compared with the controls, similar to previous studies in human participants with amygdala lesions^16^,^17^. A role for the amygdala in driving exploration of the eyes is further substantiated by neuroimaging and electrophysiological evidence. For example, amygdala activation is increased when human subjects direct saccades to the eye region^31^, and neurophysiological recordings have shown that the non-human primate amygdala contains neurons that respond selectively when fixating the eye region^18^.

When we examined changes in pupil diameter during free viewing of different facial expressions, increased pupil dilation when viewing threat faces was evident in monkeys with and without amygdala lesions. This result is consistent with a recent study that showed greater pupil dilation when monkeys viewed threat compared with other facial expressions^20^. Pupil dilation has been interpreted as a general indicator of increased vigilance, arousal and interest, indexing behavioural responses^32^. In humans pupil dilation is largest when viewing emotionally arousing content^33^,^34^. While no prior studies in humans have examined the effect of amygdala lesions on pupillary changes while subjects viewed emotional stimuli, human patients with amygdala lesions report being less aroused when viewing negative emotional scenes^35^, and have reduced autonomic activation during emotional perception^36^. The monkeys with amygdala lesions did not show reduced pupil dilation to threat. Rather, they showed increased pupil constriction stemming from the pupillary light reflex elicited by presentation of any face, compared with controls. This might reflect reduced parasympathetic drive consistent with prior reports that stimulation and lesions of the amygdala modulates parasympathetic orienting responses^37^,^38^. These results do not necessarily contradict prior studies in humans that link autonomic arousal and perceptual vigilance to amygdala function. The activity of related brain regions, most notably the anterior insula and cingulate cortex, are known to be correlated with arousal related changes in autonomic activity in monkeys^39^, and humans^40^. Our finding of intact threat related modulation of pupil size in animals with amygdala lesions suggests autonomic arousal during socioemotional processing is not solely under the influence of the amygdala, and instead likely reflects the activity of additional brain regions implicated in autonomic control.

Thus, like the results from the attentional capture task, our results from the free-viewing task suggest that regions outside the amygdala contribute to both exploration of faces and autonomic responses to biologically relevant stimuli. In addition to the cortical areas discussed above, recent work in rodents has emphasized the importance of projections from the central nucleus of the amygdala to the ventral hippocampus, paraventricular nucleus of the thalamus, and periacqueductal gray in mediating conditioned threat responses^41^,^42^,^43^. The paraventricular nucleus and periacqueductal gray are of particular interest given that our experiments examined oculomotor behaviour, and both structures are known to project to the superior colliculus in monkey^44^, and show heightened activation during reward and fear conditioning^42^. Also in rodents the pre-limbic cortex is shown to be important for expression of threat responses^43^, particularly after threat associations have been learned. Thus, the effects we observed on attentional capture might result from disrupting sub-cortical pathways through which the amygdala modulates oculomotor circuits, whereas the effect of the lesions in the free-viewing task might stem from increased reliance on cortical structures to motivate attention to specific facial features.

Although our results have shown differences between monkeys with amygdala lesions and unoperated controls, we also found that both groups showed a similar behavioural pattern. In the first task we found that both groups of monkeys were slower to saccade to a peripheral target if presented with a mouth depicting a threat expression. When the same subjects were tested in the free-viewing paradigm we found that they spent more time exploring the eye region and they looked relatively less at the eyes and longer at the mouth if the image depicted a threat expression compared with other emotions. Thus, the amygdala lesion did not eliminate all influences of the threat stimuli, but it did decrease detection and modulated responses to these stimuli compared with controls. Previous neuroimaging studies in monkeys have shown that selective lesions of the amygdala reduce differential responses to emotions in face processing regions of the inferior temporal cortex, but these regions still respond more strongly to faces than they respond to objects^45^. Consistent with this, Tsuchiya *et al.*^46^ tested subject S.M. on rapid discrimination of fear and angry faces from neutral faces. The authors reported that S.M. showed normal rapid discrimination of these stimuli, but as reported previously^4^, S.M. rated the intensity of fear stimuli lower than did the controls^46^.

Thus the data from our study, together with the finding that the subject S.M. failed to assign normal intensity ratings to facial expressions, leads to the suggestion that the primary deficit in our monkeys with amygdala lesions, as well as in S.M., consists of a specific insensitivity to the facial expressions of emotion that convey a potential threat. Our data also revealed a role for the amygdala in driving exploration of the eye region. The extent to which the eye region of the face contributes to identification of an individual conspecific or conveys information regarding age, sex, reproductive or social status is not known. However, certain social cues are known to be highly valued, as evidenced by monkeys' sacrificing juice rewards^47^ or delaying food retrieval^48^ to view images containing information about social and reproductive status. We suggest that the amygdala may be attributing value to social stimulus features, such as eye gaze^18^. This value may then drive information that is beneficial to social interactions. Future studies should examine amygdala contributions to specific indicators of social status.

In the free-viewing paradigm both controls and amygdala lesion monkeys spent more time looking at the eyes relative to other features, even though the preference for the eyes was strongly attenuated in animals with amygdala lesions. Both groups also viewed the mouth in the threat expression more than the mouth in other expressions in the free-viewing task, which is consistent with the attentional capture effects. In the attentional capture task, however, the eyes did not slow reaction times for either group. There are several possible explanations for this discrepancy. First, the two paradigms might not engage completely overlapping neural mechanisms. In the attentional capture task, viewing the mouth came at the expense of reward rate, whereas in the free-viewing task, the reward rate was not affected by the pattern of fixation. The two tasks also unfolded on different time scales. The reaction time effects in the attentional capture task occurred over a few hundred milliseconds, whereas the animals had 1,500 ms in the free-viewing task. The longer time scales in the free-viewing task may have brought complementary neural systems—for example frontal–parietal attentional systems—into play. Further, the eyes may become more salient when they are presented in the context of the whole face to infer, for example, the emotional state or social status of a conspecific, whereas they may provide less useful information when they are presented in isolation.

Moreover, the amygdala is composed of multiple nuclei with different functions. For example, a monkey fMRI study^10^ found that the basolateral amygdala responded differentially to facial expressions (stronger response for threatening than affiliative expressions), whereas the central nucleus responded differentially to the direction of eye gaze (stronger response for averted than directed-gaze faces). Because we studied monkeys with lesions of the entire amygdala, an important question for future studies will be to determine the specific contribution of specific nuclei within the amygdala in facial emotion processing.

In our study we tested four monkeys with focal bilateral, excitotoxic amygdala lesions. Each animal was tested in several sessions and each session used a large set of unique stimuli to minimize habituation. This approach allowed us to generate a large data set on animals with highly specific lesions. We found reduced attentional capture by threat stimuli in monkeys with amygdala lesions. We also found that the animals with amygdala lesions spent less time viewing the eyes and more time viewing the mouth than control animals. Finally, threat relative to all other expressions elicited greater pupil dilation in both monkeys with amygdala lesions and controls, despite an overall reduction in pupillary light reflex in the lesion group.

One of the limitations of this study is the small sample size used. Thus, negative findings should be interpreted cautiously. However, our results are generally consistent with previous work in participants with amygdala lesions, many of which are based on data from participant S.M. Both of our studies supported the causal contribution of the amygdala in detecting threat signals, and the free-viewing task further supports a role for the amygdala in directing attention to the eye region. Thus, the amygdala does not appear to be essential for discriminating threat from other emotions or for directing gaze to the eye region of a conspecific, but instead it might play a key role in enhancing processing of salient signals by allocating more attention to them, or more generally assigning values to the biologically relevant cues as well as to the threats that surround us in everyday life.

## Methods

### Subjects

Eight adult male rhesus monkeys (*Macaca mulatta*) served as subjects. Four monkeys were given bilateral excitotoxic lesions of the amygdala (group amygdala lesion), and four were retained as unoperated controls (group control). Animals weighed between 7 and 11.5 kg at the start of testing. Each animal was housed individually, was kept on a 12-h light/dark cycle, and had access to food 24 h per day and controlled access to water during testing. All procedures were reviewed and approved by the National Institute of Mental Health Animal Care and Use Committee.

### Surgery

At the time of surgery, anaesthesia was induced with ketamine hydrochloride (10 mg kg^−1^ im) and maintained with isoflurane (1.0–3.0%, to effect). The animals received isotonic fluids via an intravenous drip. Aseptic procedures were employed. Heart rate, respiration rate, blood pressure, expired CO_2_ and body temperature were monitored throughout the procedure. After the injections of excitotoxin into the amygdala were completed, the wound was closed in anatomical layers. All monkeys received a pre- and postoperative treatment regimen consisting of dexamethasone sodium phosphate (0.4 mg kg^−1^) and cefazolin antibiotic (15 mg kg^−1^) for 1 day before surgery and 1 week after surgery. For 2 days, the monkeys received the analgesic ketoprofen (10/15 mg). Ibuprofen (100 mg) was provided for 5 additional days.

### Amygdala lesion by ibotenic acid injection

We used the same method previously described by Baxter *et al.*^49^; injections of ibotenic acid were placed stereotaxically throughout the amygdala with coordinates determined from magnetic resonance imaging scans performed before surgery. The amygdala injections were carried out in two stages (balanced for hemisphere of first surgery) separated by a minimum of 2 weeks. After the bilateral amygdala lesion was complete, monkeys were allowed to recover for 10–14 days.

After induction of anaesthesia, monkeys were placed in a stereotaxic frame. A large bone flap extending over the midline was taken in the appropriate portion of the cranium, and a final reading taken on the position of the sagittal sinus, which served as the landmark for calculation of stereotaxic coordinates in the mediolateral dimension. Slits were cut in the dura to allow passage of the injection needle. A mean of 18 injections (range 13–22), each consisting of 0.6–1.0 μl ibotenic acid (10–15 μg μl^−1^; Biosearch Technologies or Sigma), were made into the amygdala via the 30-gauge needle of a Hamilton syringe held in a micromanipulator. The injection sites were roughly 2 mm apart in each plane. Each injection was made at the rate of 0.2 μl min^−1^, and the needle was left in place 2–3 min after each injection to limit diffusion of the toxin up the needle track. The intended lesion included the entire amygdala, including both the basolateral nuclear group and the central, medial and cortical nuclei.

### Assessment of the lesions

The lesions in all four operated monkeys were quantitatively assessed from postoperative MRI scans. The extent of amygdala damage was evaluated from T2-weighted scans obtained within 10 days of surgery. For each operated animal, MR scan slices were matched to drawings of a standard rhesus monkey brain at 1-mm intervals. Each lesion was subsequently plotted onto the standard sections. The location and extent of the amygdala lesions are shown in Fig. 1.

### Head post implantation

Before testing began, and a minimum of 60 days after the amygdala lesions had been completed, each monkey received a surgically implanted head post for head fixation to allow for accurate video tracking of eye movements. Anaesthesia and physiological monitoring procedures were the same as those used for the amygdala lesions. Monkeys were allowed an additional 30–40 days of recovery after the implant surgery before training began.

### Behavioural tasks

For the first experiment monkeys were tested on an oculomotor saccade task. On each trial the monkeys were required to fixate on a cross at the center of a computer monitor for 500 ms. Then an image replaced the fixation point, while a peripheral saccade target was simultaneously presented at 9.5 degrees of visual angle. Peripheral saccade targets were randomly presented on either the left or right of central fixation. The animal's task was to saccade to the peripheral target and then maintain fixation for 500 ms to earn a juice reward.

There were two conditions that used either social distractors (intact portions of monkey faces) or nonsocial distractors (scrambled portions of monkey faces). As shown in Fig. 2a, images for the social condition consisted of portions of monkey faces (eyes, nose or mouth) extracted from face images displaying different, well-characterized expressions of emotion. The stimuli replacing the fixation target measured 47 × 21 mm. The nonsocial condition was run in the same way with the exception that the stimuli replacing the fixation cross were scrambled versions of the facial feature images. Using both intact and scrambled images of parts of conspecific faces allowed us to control for overall differences in reaction time between biologically relevant and irrelevant stimuli presented at fixation. An eye-tracking system (Arrington ViewPoint) recorded eye movements while monkeys performed the task. Stimuli were displayed on a computer monitor placed 40 cm in front of the monkey. Reaction time and accuracy were recorded. The reaction time is the time that the animal took to initiate a saccade to the peripheral target. We defined accuracy as the percentage of trials on which the monkeys successfully acquired and held fixation on the peripheral target.

We collected five sessions per animal and a maximum of 480 correct trials in each session. A session was typically under 20 min in duration. Each daily session used a different stimulus set. Each stimulus set was balanced across expression and was comprised of 480 unique images (80 eyes, 80 noses, 80 mouths, 80 eyes scrambled, 80 noses scrambled, and 80 mouths scrambled). The scrambled images were generated by taking the two-dimensional fourier transform of the original images, scrambling the phase and then taking the inverse fourier transform. This preserves the second order statistics of the images, while destroying all higher-order statistics. In each set there were four different facial expressions: neutral, submissive (also known as bared-teeth or fear grin), threat (open-mouthed threat) and affiliative (lip smack). The open-mouthed threat is used mostly in aggressive behaviours to intimidate a conspecific. The bared-teeth display is a submissive and fearful behaviour typically shown by a subordinate to a dominant animal. The lip smacking face represents affiliation. Thus, each day monkeys were presented with 20 neutral, 20 submissive, 20 threat and 20 affiliative images of each of the three-face parts (eyes, nose and mouth).

Additionally, the same subjects were evaluated in a second task that was a free-viewing paradigm. The monkeys first acquired and held a central fixation point for 1,000 ms, and then an image of a conspecific was shown for 1,500 ms depicting one of four ecologically relevant facial expressions—neutral, submissive, threat and affiliative. During the 1500-ms period monkeys were free to explore, or not explore, each face presented. At the end of the 1500, ms presentation a juice reward was delivered, regardless of the gaze pattern of the subject (Fig. 2b). Images were presented randomly in one of two different vertical positions on the screen: either the eyes or the mouth were centred at the level of the fixation point, thus balancing which facial feature was first seen by the monkeys. Valid trials are defined as those in which the monkey successfully fixated on the initial fixation point for 1,000 ms. If the monkey broke fixation during that required fixation time, the trial was counted as incorrect and no face image appeared. We included all successful trials even if the animals did not look once at the face image displayed after the initial fixation was completed. We collected five sessions per animal with a maximum of 160 valid trials per session and the duration of the session never exceeded 20 min. Each daily session used a different stimulus set. Each stimulus set was balanced across expression and was comprised of 80 unique images and four different facial expressions: 20 neutral, 20 submissive, 20 threats and 20 affiliative. All stimuli were embedded in a grey oval mask as background to remove any extraneous background features of the original photo that could be distracting.

### Data analysis

As there was no *a priori* estimate of effect size, sample size was based on a commonly accepted number of subjects for primate lesion studies. This allows for overall comparison with previous studies. As the number of animals was small, we repeated the experiment multiple times in each animal to increase the number of sessions available for statistical analysis. In the first experiment we examined whether the amygdala lesion affected reaction time (log-transformed reaction time; dependent variable) via an omnibus mixed-effects ANOVA that specified group (amygdala lesion and control), stimulus type (social and nonsocial), emotion (threat, neutral, affiliative, and submissive), and face feature (eyes, nose and mouth) as fixed factors and session number (1–5) as a random effect nested under monkey (8 subjects), which was nested under group. Second, we investigated whether the reaction time (dependent variable) was affected by stimulus type. We ran two independent ANOVAs, one for social stimuli and one for nonsocial stimuli, that specified group (amygdala lesion and control), emotion (threat, neutral, affiliative and submissive), and face feature (eyes, nose, and mouth) as fixed factors and session number as a random effect nested under monkey, which was nested under group. Third, for the social condition (intact images) we ran three separate ANOVAs for the three-face features presented in the task; one for the eyes, one for the nose and one for the mouth. Overall reaction time effects that differ by monkey were controlled for by the random effects factors in the ANOVAs. When a significant effect of emotion was found, we investigated this further by running direct *post hoc* comparisons with two-tailed independent *t*-tests and the *P*-value was Bonferroni corrected for the number of comparisons. Finally, to examine overall attentional engagement, we ran an omnibus mixed-effects ANOVA with accuracy as dependent variable, group (amygdala lesion and control), stimulus type social and nonsocial, emotion (threat, neutral, affiliative, and submissive) and face feature (eyes, nose and mouth) as fixed factors and session number as a random effect nested under monkey which was nested under group; as described above for reaction time.

With the second task we examined whether the amygdala lesion effected exploration of conspecific faces depicting one of four ecologically relevant facial expressions—neutral, submissive, threat and affiliative. We delineated three AOIs to quantify the amount of attention the monkeys directed towards the eyes, nose and mouth. For each animal the total looking-time was calculated using software written in Matlab (Math Works, Natick, MA, USA) that calculated the total time that the animals spent within the boundaries of the three AOIs. We normalized data within trials to control for individual differences and variations in number of fixations across test days^50^. The proportion of time fixations made within the eyes or nose or mouth regions were normalized by the total looking-time spent within the entire face region on each trial. All analyses were computed using normalized data. We investigated whether the amygdala affected the proportion of looking-time (dependent variable) in the three-face region AOIs: eyes, nose and mouth. For the dependent measure we calculated a mixed-effects ANOVA model specifying group (amygdala lesion and control), face position (eyes centred and mouth centred), emotion (neutral, submissive, threat and affiliative) and regions (eyes, nose and mouth) as fixed factors and session number as a random effect nested under monkey. For each analysis direct *post hoc* comparisons were made with two-tailed independent *t*-tests and the *P*-value was Bonferroni corrected for the number of comparisons.

Additionally, we examined whether amygdala lesion affected changes in pupil size in the free-viewing task. Pupillary changes were first baseline corrected on a trial-by-trial basis by subtracting the mean change in pupil diameter 500 ms before stimulus presentation, the period when the animal stably fixated the center of the screen. Outliers related to lid closure or loss of the eye-tracking signal (<1% of all samples) during the free-viewing period were detected and linearly interpolated over using the nearest valid, adjacent samples. Stimulus driven changes in pupil diameter were then averaged across trials contributing to each condition. Based on visualization of the average response profile the mean change in pupil diameter was extracted for two non-overlapping time windows per session. The first 250–750 ms window captured the magnitude of the initial light reflex, whereas the second window from 750–1500, ms indexed pupillary dilation during free viewing. Effects on pupil change within each time window were separately tested via mixed-effect ANOVAs that specified group (amygdala lesion and control), initial face position (eyes centred and mouth centred) and emotion (neutral, submissive, threat and affiliative) as fixed factors.

## Additional information

**How to cite this article:** Dal Monte, O. *et al.* Amygdala lesions in rhesus macaques decrease attention to threat. *Nat. Commun.* 6:10161 doi: 10.1038/ncomms10161 (2015).
